# Badminton Improves Executive Function in Adults Living with Mild Intellectual Disability

**DOI:** 10.3390/ijerph20043673

**Published:** 2023-02-19

**Authors:** Yifan Wang, Xueping Wu, Huawei Chen

**Affiliations:** 1 College of Physical Education, Shanghai University of Sport, Shanghai 200438, China; 2 Sports Department, Nanjing University of Aeronautics and Astronautics, Nanjing 210016, China

**Keywords:** mild intellectual disability, badminton, executive function, adult, exercise rehabilitation

## Abstract

Background: Adults with intellectual disability have limited executive function—which includes working memory, cognitive flexibility, and inhibitory control subcomponents—making their ability to live independently challenging. The present study explored whether a badminton intervention program could improve the executive function of adults living with a mild intellectual disability, but with no physical disability. Methods: This randomized controlled study randomly assigned 30 adults with mild intellectual disabilities recruited from Shanghai Sunshine bases in Shanghai (20 males and 10 females; mean age, 35.80 (3.93) years) to a badminton intervention program (*n* = 15, training for 12 weeks, 3 times/week, 60 min each time) or the control group (*n* = 15), which received a typical physical education course consisting primarily of gymnastics. Correct response rates and response times on the Stroop test, n-back task, and task switching were analyzed using two-way analyses of variance, followed by simple effects tests to evaluate inhibitory control, working memory, and cognitive flexibility, respectively, before and after the badminton intervention. Results: No significant difference was detected between the badminton group and the control group (*p* > 0.05) for their pre-test scores on any subcomponent of executive function. A 2 × 2 repeated-measures analysis of variance showed a significant increase in accuracy in the inhibitory control task for the badminton group after the intervention (*p* < 0.05). In addition, the accuracy rate and reaction time in a working memory task were significantly improved in the badminton group after the intervention (*p* < 0.05). Although some improvement in cognitive flexibility was observed for this group after the intervention, it was not statistically significant (*p* > 0.05). In the control group, there was no significant difference in any executive function subcomponents after the intervention (*p* > 0.05). Conclusions: These results suggest that badminton may be used as an effective intervention to improve the executive function of adults with a mild intellectual disability and that our protocol may inform future badminton exercise intervention programs.

## 1. Introduction

Executive function refers to the efficient, orderly, and purposeful behaviors of individuals who process and coordinate cognitive links during complex cognitive activities to achieve specific goals [1]. Executive function comprises three subcomponents: inhibitory control, working memory, and cognitive flexibility [2,3,4]. The three components are related to and are independent of one another [5]. Executive function continues to develop from childhood to adulthood and are essential for adaptive behavior [6]. Deficits in executive function development lead to poorer inhibitory control, working memory, and cognitive flexibility in individuals. It may also lead to the production of low adaptive behavior in life [7].

Intellectual disability is a functional disability characterized by significant limitations to intellectual functions and adaptive behaviors [8,9]. The prefrontal lobe of the brain is associated with executive function. However, during growth and development, the brain structures and functions of people with intellectual disability become different from those of people without this disability because of the alterations and obstacles to their physical function, with studies showing cerebral cortical dysplasia, prefrontal lobe damage, and other brain structural abnormalities [10,11,12,13]. Chevalere measured the executive function of 17 mentally disabled individuals with Pradwilli syndrome compared with that of 17 healthy individuals. The results showed that the inhibitory, refreshing, and switching functions of the mentally disabled individuals were significantly impaired compared with those of the able-bodied individuals [14]. In such adults, the entire brain or the hippocampus or temporal lobe may be reduced in volume, limiting their executive function. These adults may have the ability to understand communication, but pathological abnormalities may cause poor social adaptation abilities, making independent living extremely challenging.

There is a close relationship between exercise and executive function. It has been well documented that healthy physical fitness is positively correlated with executive function, and all components of healthy physical fitness are related to executive function. Frost conducted tests of cardiopulmonary function and executive function in 99 elderly people and concluded that the higher the level of cardiopulmonary fitness was, the better the executive function was [15]. Li Yue selected 100 college students as the research objects and tested their healthy physical fitness and executive function. The results showed that the ability of all aspects of healthy physical fitness of the college students was related to executive function, and the higher the overall level of healthy physical fitness was, the better the performance of executive function tasks was [16]. This suggests that engaging in regular physical activity is necessary to enhance the development of executive function in the human body. For now, the use of exercise intervention is becoming a mainstream approach to improve executive function, and many studies have shown that physical exercise has a positive impact on the executive function of people with intellectual disabilities who do not have any physical impairments [17,18]. Through a comparative study of different exercise methods on executive function, it was found that open skills exercise combined with different or multiple physical activities with moderate intensity enhanced all of the components of executive function better [19,20,21,22].

Badminton is an open skills sport. It requires participants to select appropriate actions over a short time to return the shuttlecock over the net to the opposing team by various hitting and footwork techniques. Hitting the shuttlecock in different directions with varying heights and speeds can improve the excitability and sensitivity of the nervous system and increase the number of muscle fibers involved in the sport. These characteristics have been shown to improve the executive function and physical function of people who have played badminton for a long time [23,24]. In addition, badminton is suitable for both younger and older persons. The game is interesting and confrontational, which not only provides the participants with entertainment, but it also promotes communication between them. Some previous studies have shown that badminton is highly likely to improve human executive function. In a study investigating the effects of physical activity on executive functioning in disabled athletes, Russo compared the executive functioning in disabled basketball players, disabled swimmers, and healthy non-athletes. The results indicate that the brain components associated with performing processing tasks were impaired in the disabled athletes. However, open sports such as basketball may be able to compensate for a part of the impairment in executive function by fostering stability in motor responses and supporting flexibility in response. Therefore, compared with the closed exercise, the open exercise has a better effect on the promotion of human executive function. A study by Yu and Zhao found that moderate-intensity badminton improves executive function—including the subcomponents of inhibitory control, working memory, and cognitive flexibility—among adults, and the effect is related to the length of time performing the exercise, with longer times associated with greater improvements in executive function [24].

However, the effect of badminton on the executive function of adults living with intellectual disability is unclear owing to inconsistent results among the existing studies. Therefore, the present randomized, controlled study explored whether 12 weeks of badminton skills training improved the executive function of adults with a mild intellectual disability, but no physical disability. According to the relevant article by Michal, it is suggested that comprehensive rehabilitation elements are effective in practice. The prosocial behavior level of people with an intellectual disability is low, and comprehensive methods should be paid attention to in the process of intervention. Interventions that strictly follow the individual characteristics of the subjects have a higher probability of success [25], not only to improve their motor skills and cognitive abilities, but also to provide support to achieve the highest educational level in the intervention [26]. Therefore, the intervention curriculum for persons with an intellectual disability was developed with a comprehensive consideration of their physical and mental health. Additionally, strict attention was paid to each person’s physical condition and acceptance ability during the intervention to provide them with personalized intervention methods to the greatest extent.

## 2. Materials and Methods

### 2.1. Participants

Adults with mild intellectual disabilities were recruited for this randomized controlled study from the Sunshine bases located in various areas of Shanghai. The study was conducted from August to December 2021. We recruited males and females who met the following criteria: (1) an intelligence quotient as assessed by the mental health centers of between 50 and 69, with a disability certificate; (2) 30–40 years of age; (3) no previous badminton training; (4) no history of chronic disease (e.g., heart disease or epilepsy); (5) no physical disorder. Persons with the following conditions were excluded: (1) attention-deficit/hyperactivity disorder and those unable to engage in regular physical activity; (2) severe cardiovascular disease or history of severe trauma. All of the participants and their parents or guardians provided written informed consent prior to the beginning of this study.

A total of 30 participants (20 males and 10 females) were included in this study. The participants each received a number from 1 to 30 that was randomly generated by Microsoft Excel, RANDBETWEEN. The participants were assigned to either the experimental group (numbers 1–15) or the control group (numbers 16–30), with 15 people in each group. The participant baseline demographic information is given in Table 1. The mean (±standard deviation) age was 35.80 ± 3.93 years, the mean height was 168.68 ± 10.29 cm, the mean weight was 72.25 ± 14.25 kg, and the mean body mass index (BMI) was (25.05 ± 4.12) kg/m^2^. Sex differences were analyzed by Crosstabs in SPSS software, version 22.0. There were no significant sex differences in age, height, weight, and BMI (all *sig* values > 0.05).

### 2.2. Experimental Design and Measurements

#### 2.2.1. Badminton Intervention Program Development and Content

We referred to previous badminton intervention programs conducted in China in related fields that used a total of approximately 12 weeks, 2–3 times per week, 60 min each time [27,28] to set the exercise intervention times in the present study as 60 min/time, 3 times/week, for a total of 12 weeks. The training content was developed with reference to the coach’s manual for Special Olympic Functional Activities, with consideration for the mental and physical conditions and health level of people with intellectual disabilities, and after communicating with professional badminton coaches. The intervention content was arranged around the three subcomponents of executive function. For inhibitory control, the participants needed to avoid interference. When they learned and mastered different technical actions, they were able to perform correct actions through the use of the instructions provided. In addition, it was necessary to strengthen their control to improve their control ability, and suppress the generation of wrong actions. For working memory, the participants were required to constantly memorize and process information and to store actions, while learning new skills. For cognitive flexibility, the participants were required to flexibly switch between working memory states.

The badminton intervention program consisted of a preparation period that included warm-up exercises and jogging, followed by specific badminton skills training with gradually increasing duration and intensity, and it ended with a static stretching period (Table 2). The exercise intensity was monitored. After each exercise session, 6 participants were randomly selected, and their heart rates were assessed manually, and their observed physical states were recorded by their supervisor. During the skills training period, the mean heart rate of the participants was 135.74 beats per minute, which was equivalent to 65–75% of the maximum heart rate for this age group, indicating moderate-intensity exercise.

The badminton skills training included forehand and backhand racket holding, forehand and backhand serving, hitting a shuttlecock high, net setting, net picking, and hitting a shuttlecock high before the net caught it. Training also included comprehensive practice and was supplemented by physical quality exercises necessary for badminton practice. The control group maintained their original working, living, and exercise habits in the Sunshine base during the experiment. Members of the Sunshine base, led by specific teachers, attended regular 60 min physical activity sessions 3 times/week without any other intervention.

#### 2.2.2. Measurement of Executive Function

The Stroop test, n-back task, and task switching were used to evaluate inhibitory control, working memory, and cognitive flexibility, respectively, of adults with a mild intellectual disability before and after the badminton intervention [29,30,31,32].

Inhibitory control was measured using the Stroop test. The Stroop color task was divided into consistent and inconsistent conditions. In the consistent condition, the word presented by the test instrument was the same as its color, and in the inconsistent condition, the word did not match the color. The participants needed to pay more attention to judge the words and avoid choosing the incompatible colors. Working memory was measured using an n-back test, as designed by Aiguo and colleagues [33]. In this test, the working memory is continuously refreshed and monitored. Cognitive flexibility was measured using a task switching test. A two-task (with shapes and colors), two-level (with blue and orange; squares and circles), simple task-cueing paradigm was used for testing. Based on the content to be judged, it can be divided into three conditions (codes). A test of identifying shapes or colors alone reflects a general visual judgment, whereas tasks in which different contents are judged according to the instructions reflect a task switching ability.

The tests were conducted using E-prime 3.0 software. The participants responded to the stimulus pictures presented by E-prime software, and the software automatically collected the task data. The preprocessing of the executive function data was also completed by the software. The evaluation of executive function included the correct response rate and response times. A faster response time indicated a better executive function. The accuracy (rate of correct responses) was used as a secondary evaluation, with greater accuracy being represented by a higher score.

### 2.3. Statistical Analysis

SPSS 22.0 software was used for the statistical analyses. The Kolmogorov–Smirnov test was performed on the indicators of executive function assessed before the badminton intervention in the two groups of adults with a mild intellectual disability. All of the indicators in both groups were normally distributed. Independent-samples *t* tests were used to determine whether there were differences between the badminton group and the control group at the baseline. Two-way repeated-measures analyses of variance (ANOVAs) were used to assess data from the badminton and control groups (between group factor) and from the badminton group before and after the intervention (i.e., time; within group factor), as well as the effect of time by each group. Significant interactions were further explored using simple effects tests. An α of 0.05 was considered to be statistically significant.

## 3. Results

### 3.1. Differences in Executive Function between the Badminton and Control Groups before Badminton Intervention

Independent-samples *t* tests were used to analyze the differences for each index of executive function. The results showed that there were no significant differences in the indexes of inhibitory control, working memory, and cognitive flexibility between the badminton and control groups before the exercise intervention (*p* > 0.05) (Table 3), indicating that baseline levels of executive function were similar between the two groups before the intervention.

### 3.2. Differences in Executive Function before vs. after Badminton Intervention

After 12 weeks of badminton intervention, the indexes of executive function as assessed by the inhibitory control, working memory, and cognitive flexibility scores of the participants in the badminton intervention vs. the control group had changed. During this process, the accuracy of inhibitory control and working memory level were significantly improved.

#### 3.2.1. Inhibitory Control

Multivariate repeated-measures ANOVAs were performed to assess the changes in the inhibitory control indicators as assessed in the Stroop task between the two groups of participants (badminton vs. control) over time (before vs. after intervention) (Table 4). There was a significant main effect of time for the participant response time in the inconsistent condition of the Stroop task. For the response time in the consistent condition, there was a significant main effect of time. For accuracy in the inconsistent condition, there was a significant main effect of time and time by group interaction. For accuracy in the consistent condition, the main effect of group was statistically significant, and the main effect of time and the time by group interaction were also significant.

**Figure 1:**
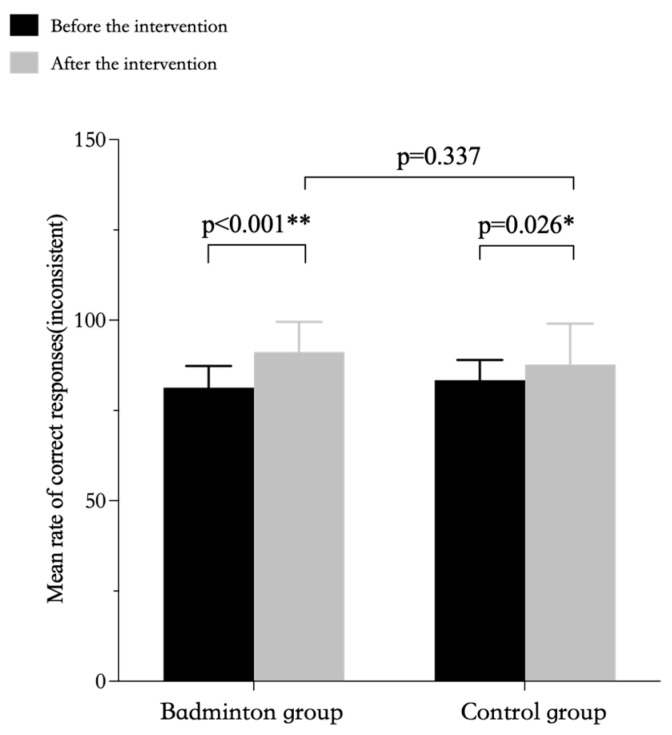
Rates of correct responses in the inconsistent condition of the Stroop test by group. * *p* < 0.05, representative *p* values have significant differences. ** *p* < 0.01, represents *p* values with extremely significant differences.

**Figure 2:**
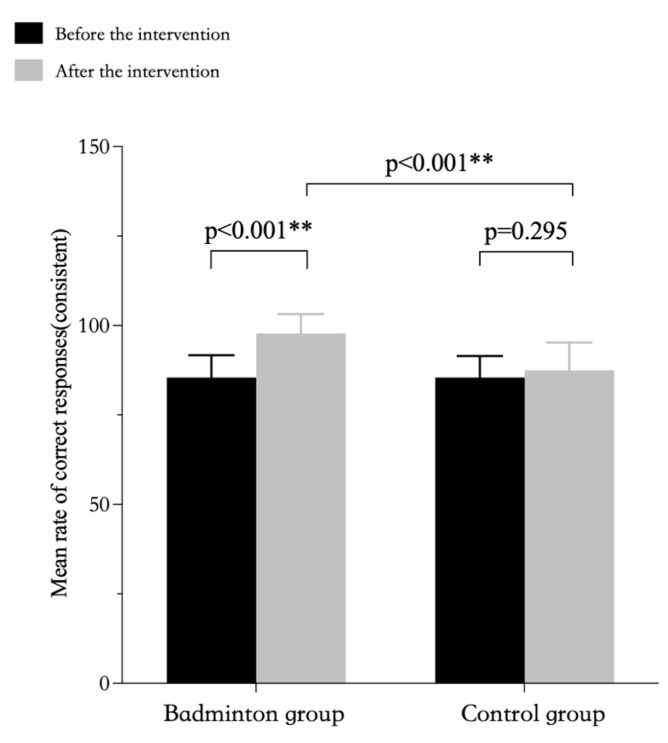
Rates of correct responses in the consistent condition of the Stroop test by group. ** *p* < 0.01, represents *p* values with extremely significant differences.

The repeated-measures ANOVA results showed a significant interaction for accuracy in both conditions between the badminton intervention group and the control group. The results of the simple effects analyses showed that in terms of accuracy (inconsistent condition), there was a significant difference between the pre-intervention and post-intervention Stroop test results and the pre-intervention and post-intervention test results in the control group for the badminton group over time, but the post-intervention test results between the two groups were not statistically significant (Table 5, Figure 1). The results of the simple effects analyses showed that in terms of accuracy (consistent condition), there was a significant difference between the pre-intervention and post-intervention Stroop test results and the post-intervention test results between the two groups for the badminton group over time, but the pre-intervention and post-intervention test results in the control group were not statistically significant (Table 5, Figure 2).

Time within group 1: the comparison of the results measured by the Badminton group at the pre-intervention and post-intervention time points; Time within group 2: the comparison of the results measured by the control group at the pre-intervention and post-intervention time points; Group within time (2): the comparison of test results between the two groups after the intervention. The F value was used to assess the differences between the groups. Values under Time effect, Group effect, and Interaction are *p* values. * *p* < 0.05, representative p values have significant differences. ** *p* < 0.01, represents *p* values with extremely significant differences. η^2^ is the effect size.

Simple effects analysis. Correct response rate calculated by prime9 software, version 9.4.1. Error bars indicate standard deviation. The same as follows.

#### 3.2.2. Working Memory

Changes in working memory indexes as assessed in the 1-back test between the two groups were analyzed by repeated-measures ANOVAs (Table 6). For response time, we found significant differences for the main effect of time (before vs. after intervention) and the interaction between group (badminton vs. control) and time, but no significant main effect of group. Similarly, for accuracy, there were significant differences in the main effect of time and the interaction between group and time, but the main effect of group was not statistically significant.

**Figure 3:**
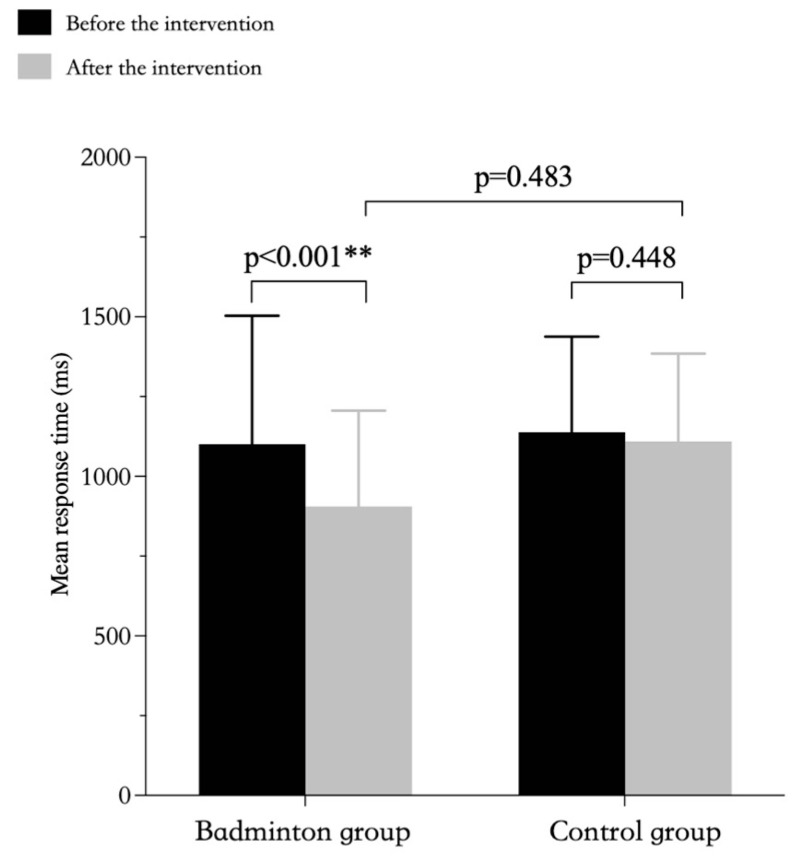
Response time in the 1-back test to assess working memory by group. ** *p* < 0.01.

**Figure 4:**
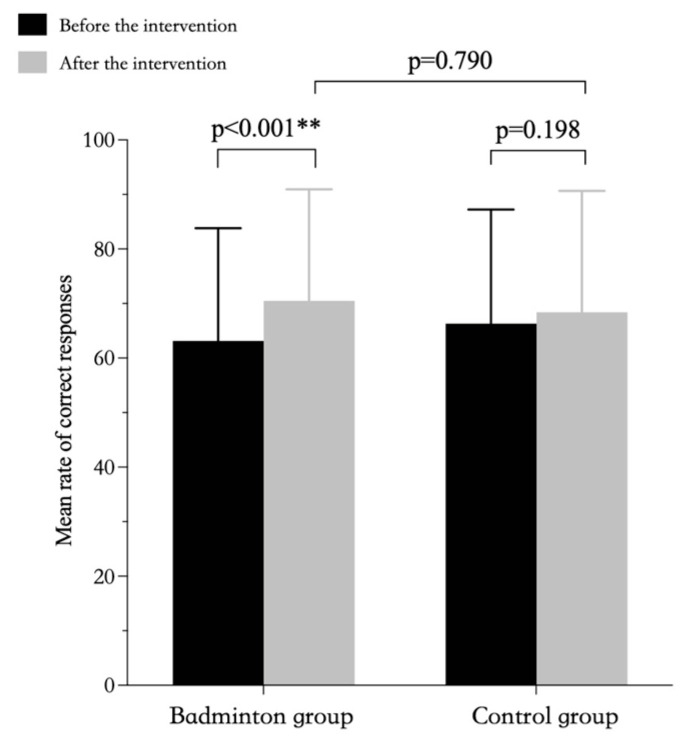
Rates of correct responses in 1-back test to assess working memory by group. ** *p* < 0.01.

The results of repeated-measures ANOVAs showed that the interactions between group and time for both response time and for accuracy were significantly different. Further simple effects analyses showed that there was a significant difference in response time between the badminton and control groups, but no significance differences before vs. after intervention for the control group nor for the post-intervention test results between the two groups (Table 7, Figure 3). In terms of accuracy, there was a significant difference in the pre-intervention and post-intervention test results in the badminton group, but no significant difference in the pre-intervention and post-intervention test results for the control group nor for the post-intervention test results between the two groups (Table 7, Figure 4).

#### 3.2.3. Cognitive Flexibility

Repeated-measures ANOVAs were performed to analyze the changes in the cognitive flexibility indicators as assessed in a task switching test for the two groups (badminton vs. control) with time (before vs. after intervention) (Table 8). We found no statistically significant main effects of time or group and no time by group interaction for response time in the trails requiring no task switching. For response time in the trials requiring task switching, there was a significant main of time, but no significant main effect of group or time by group interaction. For response time in the task switching minus no task switching condition and accuracy in either task switching or no task switching conditions, there were no significant main effects of time or group and no significant time by group interactions.

## 4. Discussion

This randomized, controlled study investigated the effects of a badminton exercise program on the executive function of adults living with a mild intellectual disability, but no physical disability. The results showed that 12 weeks of badminton skills training improved two subcomponents of executive function, inhibitory control and working memory, but not a third subcomponent, cognitive flexibility. By contrast no subcomponent of executive function among the participants in the control group that maintained their general physical activities showed significant changes.

### 4.1. Effects of Badminton on Inhibitory Control among Adults with Mild Intellectual Disability

Su Wen [28] and Ji Xiaohai [30] found that although the response time of the participants was decreased and the accuracy (rate of correct response) was improved after a badminton intervention among primary school students, the changes were not statistically significant. They believed that the reason for this was because the sensitive period for the development of inhibitory control is 6–7 years of age, and this gradually slows after the age of 10 years [34]. Some researchers also believe that the core cognitive ability of adults has been fully developed, and that their cognitive plasticity may be lower than that of children or older adults [35]. However, although the inhibitory control of adults with a mild intellectual impairment is significantly lower than that of their healthy peers, they have developed mature brain and body functions, thus an enhancement in the Stroop task response may not be evident. However, it has also been suggested that short-term, complex exercise has a better effect on promoting cognitive function, whereas longer and more intense exercise increases the cognitive fatigue caused by cognitive demands, which affects the influence of such interventions. In the present study, the participants spent a relatively long time practicing badminton (12 weeks), and the intensity of the intervention program was kept at a medium-to-high load, which may have also caused cognitive fatigue when the physical exertion was large, leading to no significant improvement in the response time of adults with mild intellectual disabilities.

In terms of accuracy, as assessed by the rate of correct responses, Yang Hong [27] found that the rate was significantly improved under two conditions after 3 months of badminton intervention among junior middle school students, which was consistent with the results of this study. However, a study assessing a badminton intervention for college students showed that although the accuracy in the Stroop task appeared to be improved, the effect was not statistically significant [36]. The reason for the lack of a significant effect in their study may be that badminton requires a lot of inhibitory control. When one is learning technical movements and footwork for badminton, the brain should inhibit the wrong movements and footwork, such as during the conversion of the forehand and backhand grips and turning the body fully to the side, with hands raised at right angles to the torso, to hit a shuttlecock high. The instructors constantly reminded and corrected participants about such details during the intervention so that they could correctly complete movements and play at a high level. In addition, once the participants were proficient in multiple technical skills, the instructor randomly called for different actions, and the participants were required to perform the correct action. Such exercises mobilized the relevant functions of the participants and engaged their inhibitory control. During play, the participants not only needed to focus on the direction, speed, and trajectory of the shuttlecock, but also, they were required to choose the appropriate footwork and style of hitting the shuttlecock based on its spatial position. Xuejun Bai found that the increase in the gray matter volume in the middle temporal gyrus of adults after continuous badminton exercise was related to them processing rich, dynamic information and accurate visual perception prediction during badminton exercise [37]. Such cognitive demands greatly activate the inhibitory control function of relevant regions in the brain and may be associated with the significant improvement in the accuracy in the Stroop task for the consistent condition observed in the present study.

### 4.2. Effects of Badminton on Working Memory among Adults with a Mild Intellectual Disability

Relevant studies conducted in China [38] have shown that the working memory is significantly enhanced after badminton exercise, a result that is similar to that of the present study. The reason for this finding may be that complex technical actions boost the working memory. Different from other sports, badminton requires that the subject can quickly receive the information in the action and can exclude the irrelevant information, leaving only the needed information and storing it in the brain for a short time [27]. For example, Guo Xiumeng [38] conducted an 8 week badminton intervention for junior middle school students and found that working memory depends on the participation of the dorsolateral prefrontal cortex and left hippocampus. Badminton skills practice may stimulate the relevant brain regions for an extended time to lead to improvements in the working memory. In badminton, players not only need to practice hand movements, but they also need to master footwork and use it appropriately to hit the shuttlecock. For instance, to move and catch a high shuttlecock in badminton, it is necessary to first turn the racket sideways, slide the foot backward, kick off and take off at the same time, turn the racket, and return to the ready position by stepping back after landing. Such a technical action contains multiple aspects, and the participants need to process and remember all of those aspects when they are executing the action. When one is learning a new technical action, the brain needs to review the previous technical action, and then absorb and sort each aspect of the next technical action one at a one. This process leads to constant refreshing of the brain’s information during the intervention to master the technical skills. A study by Aadland [39] showed that physical activity that promotes the aerobic capacity, and skills practice has a better effect on executive function than aerobic capacity does alone. During badminton sparring, participants are required to quickly interpret the information about the opponent’s shuttlecock, eliminate the wrong information, and then select the correct stroke action and memory stored in the brain to prepare for returning the shuttlecock. Because this process is constantly repeated in badminton, it may have a positive effect on the working memory in adults with a mild intellectual disability.

Research shows that the working memory of people who are approximately 7 to 10 years of age can be significantly increased [28]. Although adults with mild intellectual disabilities may have missed the sensitive period in the development of working memory because their prefrontal function and other brain functions may be reduced compared with those of healthy people of the same age, the increased use of the brain regions associated with working memory in the present badminton intervention may have led to an improved working memory.

### 4.3. Effects of Badminton on Cognitive Flexibility among Adults with a Mild Intellectual Disability

In a study that used a variety of sports as interventions among primary school students, the authors found that those who played badminton for a long time had improved cognitive flexibility and performed better than those who played other sports did [40]. Ji Xiaohai [30] showed that badminton had a positive impact on cognitive shifting among primary school students. This may be because net sports are interesting, and in badminton, the direction and speed of the shuttlecock are not consistent. During training, the participants need to constantly react to different situations based on the actions they have learned. This requires cognitive flexibility to rapidly adjust the details of the technique based on the presentation of each shuttlecock. In the intervention conducted in the present study, the participants with a mild intellectual disability underwent training to learn to switch back and forth between a high shuttlecock and net shuttlecock transfers, to pay attention to the body while they are running and the space between the racket and shuttlecock, and to perform high-speed movements in a variety of changing conditions, thus promoting improvement in cognitive flexibility. During footwork training, the instructors also set up interesting and changeable multipoint command footwork exercises. The participants were required to switch between movement techniques, and also, to perform corresponding responses based on the instructor’s gestures and commands. Thus, the results of the present study indicated that after such repeated practice, cognitive flexibility can be improved in adults with a mild intellectual disability.

However, there are different views on the improvement in cognitive flexibility associated with badminton. Some researchers assert that badminton significantly promotes cognitive flexibility after an acute intervention or after a period of time [28,41]. However, other researchers argue that badminton does not promote cognitive flexibility as well as it enhances inhibitory control and working memory [27,38]. Given the findings of the present study, the differing results of the various studies may be related to the design of the intervention program. In previous studies, long-term badminton exercise had a better effect on executive function than short-term badminton exercise did [39]. For the badminton intervention program used in the present study, the first 9 weeks were focused on fixed points and single technical movements. In the last 3 weeks, the skills learned in the first 9 weeks were combined for comprehensive practice, with sparring and competition exercises requiring high cognitive flexibility being added. However, we believe that the amount of time in which these comprehensive exercises were conducted was insufficient, such that although the switching reaction times and correct rates in the badminton group improved after the intervention, the improvement was not statistically significant.

## 5. Conclusions

This randomized controlled study found significant improvement in the executive function of adults with a mild intellectual disability after 12 weeks of a badminton intervention. This improvement in executive function was specifically reflected in enhanced inhibitory control and working memory. By contrast, the apparent improvements in cognitive flexibility were not statistically significant. These results suggest that badminton may be used as an effective intervention to improve the executive function of adults with a mild intellectual disability. However, in the practice process, attention should be paid to control the amount of exercise, and the physical changes in people with intellectual disabilities should be strictly paid to during the intervention. In the badminton intervention, more interesting and simple methods can be used to promote the understanding of the movement technology of the group, and the most suitable exercise intervention mode for the intellectual disability group can be explored. This study offers practical information to inform exercise intervention research on executive function in specific populations and potential badminton program intervention protocols.

## 6. Limitations

This study had some limitations. Future studies should increase the sample size to make the results more representative. The intervention period should be extended, and an interim test should be added to the pre-intervention and post-intervention tests to observe the changes in the intervention time in the improvement of executive function.

## 7. Contribution to the Field

Adults with an intellectual disability have a limited executive function, making their ability to live independently challenging. Physical exercise may improve certain aspects of the executive function in this population. However, whether badminton can improve executive function is unclear, owing to the inconsistent results among previous studies. This randomized controlled study found that 12 weeks of training in badminton skills and game playing improved the executive function of adults with a mild intellectual disability. The moderate-to-vigorous intensity physical activity performed during the training not only promoted executive function, but it also improved the physical health of the individuals in this population. The badminton program designed for this study can be replicated to enrich the physical activity interventions for people with an intellectual disability to strengthen their body and improve their brain function and their overall quality of life.

## Figures and Tables

**Table 1:** Demographic characteristics of the participants.

Group	Sex	Age, y Mean (SD)	Height, cm Mean (SD)	Weight, kg Mean (SD)	BMI Mean (SD)
Badminton (*n* = 15)	Men (*n* = 10)	36.00 (3.64)	168.53 (9.23)	70.51 (12.98)	24.87 (4.52)
	Women (*n* = 5)				
	Sig	0.486	0.378	0.467	0.378
Control (*n* = 15)	Men (*n* = 10)	35.60 (4.32)	170.83 (11.46)	73.98 (15.26)	25.24 (3.84)
	Women (*n* = 5)				
	Sig	0.371	0.161	0.233	0.387
Total		35.80 (3.93)	168.68 (10.29)	72.25 (14.25)	25.05 (4.12)

BMI, body mass index = weight in kg divided by height in meters squared.

**Table 2:** Badminton intervention protocol.

	Training Content	Time	Strength
Warm up	Warm-up exercises, mobile warm-up exercises, and jogging	10 min	Low and medium
Training	Badminton skills practice	40 min	Medium and high
1–3 weeks	Forehand grip, forehand grip, forehand serve, backhand grip, and backhand serve	Swing 10 min	
		With the shuttlecock 20 min	
	Quality training: badminton special footwork, plank support, and static squat	Quality 10 min	
4–6 weeks	High shuttlecock technique (place swing, jump swing, and moving swing)	Swing 10 min	
		With the shuttlecock 20 min	
	Quality training: a variety of jumping, crawling exercises, and heel exercises	Quality 10 min	
7–9 weeks	Net in situ and mobile net technique and shuttlecock picking technique	Swing 10 min	
		With the shuttlecock 20 min	
		Game 10 min	
	Quality training: running 1000 m and standing on one foot with their eyes closed	Quality 10 min	
10–12 weeks	Using a technique before catching a high shuttlecock (moving footwork practice); game practice	Swing 10 min	
		With the shuttlecock 20 min	
		Game 10 min	
	Quality training: full command footwork, push-ups and full squats	Quality 10 min	
Cool down	Static stretching	10 min	Low

**Table 3:** Executive function by experimental group before badminton intervention.

Executive Function	Test Condition	Badminton (*n* = 15)	Control (*n* = 15)	t	*p*
Stroop test		Response time (ms)			
	Inconsistent	712.42 (163.15)	740.12 (132.83)	0.51	0.614
	Consistent	695.04 (172.77)	723.60 (156.39)	−0.475	0.639
	Inhibitory control ability	−28.35 (85.05)	−23.48 (198.10)	0.088	0.931
		Accuracy (% correct)			
	Inconsistent	81.33 (6.01)	83.38 (5.62)	−0.964	0.343
	Consistent	85.48 (6.20)	85.48 (6.02)	0.209	0.836
1-Back test		Response time (ms)			
		1100.84 (402.60)	1138.58 (299.18)	−0.998	0.335
		Accuracy (% correct)			
		63.13 (20.67)	66.32 (20.92)	−1.279	0.222
Task switching		Response time (ms)			
	Trails requiring no task switching	1152.44 (181.10)	1127.04 (200.76)	0.364	0.719
	Trials requiring task switching	1429.03 (284.20)	1483.10 (322.77)	0.487	0.630
	Cognitive flexibility ability	276.60 (152.81)	356.05 (184.96)	−1.283	0.210
		Accuracy (% correct)			
	Trails requiring no task switching	85.72 (11.76)	81.25 (13.78)	0.955	0.348
	Trials requiring task switching	73.77 (19.52)	71.67 (17.19)	0.350	0.732

**Table 4:** Inhibitory control scores by experimental group after badminton intervention.

Executive Function	Stroop Test	Badminton (*n* = 15)		Control (*n* = 15)		Time Effect	Group Effect	Interaction
		Before Intervention Mean (SD)	After Intervention Mean (SD)	Before Intervention Mean (SD)	After Intervention Mean (SD)			
Inhibitory control	Response time, inconsistent (ms)	712.42 (163.15)	618.57 (140.30)	740.12 (132.83)	704.12 (159.78)	0.002 **	0.278	0.137
	Response time, consistent (ms)	695.04 (172.77)	628.38 (153.84)	723.60 (156.39)	701.08 (157.45)	0.024 *	0.369	0.247
	Response time. inconsistent/consistent	−28.35 (85.05)	−9.81 (90.54)	−23.48 (198.10)	3.03 (134.24)	0.433	0.827	0.889
	Accuracy, inconsistent, (%) correct	81.33 (6.01)	91.22 (8.28)	83.38 (5.62)	87.67 (11.39)	0.000 **	0.782	0.039 *
	Accuracy, consistent, (%) correct	85.48 (6.20)	97.81 (5.37)	85.48 (6.02)	87.48 (7.77)	0.000 **	0.021 *	0.000 **

Values under Time effect, Group effect, and Interaction are *p* values. * *p* < 0.05, representative *p* values have significant differences. ** *p* < 0.01, represents *p* values with extremely significant differences.

**Table 5:** Simple effect analysis of Inhibitory control scores.

Indicators	Sources of Variation	F	*p*	η^2^
Stroop test accuracy, inconsistent	Time within group 1	29.347	0.000 **	0.512
	Time within group 2	5.512	0.026 *	0.164
	Group within time (2)	0.953	0.337	0.033
Stroop test accuracy, consistent	Time within group 1	79.494	0.000 **	0.740
	Time within group 2	1.141	0.295	0.039
	Group within time (2)	19.627	0.000 **	0.412

* *p* < 0.05, representative *p* values have significant differences. ** *p* < 0.01, represents *p* values with extremely significant differences.

**Table 6:** Working memory scores by experimental group after intervention.

Executive Function	1-Back Test	Badminton (*n* = 15)		Control (*n* = 15)		Time Effect	Group Effect	Interaction
		Before Intervention Mean (SD)	After Intervention Mean (SD)	Before Intervention Mean (SD)	After Intervention Mean (SD)			
Working memory	Response time, ms	1100.84 (402.60)	905.70 (299.98)	1138.58 (299.18)	1109.24 (275.37)	0.001 **	0.488	0.001 **
	Accuracy, % correct	63.13 (20.67)	70.50 (20.44)	66.32 (20.92)	68.40 (22.26)	0.001 **	0.944	0.025 *

Values under Time effect, Group effect, and Interaction are *p* values. * *p* < 0.05, ** *p* < 0.01.

**Table 7:** Simple effect analysis of Working memory scores.

Indicators	Sources of Variation	F	*p*	η^2^
1-back test response	time within group 1	26.185	0.000 **	0.483
	time within group 2	0.592	0.448	0.021
	Group within time (2)	3.748	0.063	0.118
1-back test accuracy	time within group 1	21.701	0.000 **	0.437
	time within group 2	1.736	0.198	0.058
	Group within time (2)	0.072	0.790	0.003

** *p* < 0.01.

**Table 8:** Cognitive flexibility scores by experimental group after intervention.

Executive Function	Task switching Test	Badminton (*n* = 15)		Control (*n* = 15)		Time Effect	Group Effect	Interaction
		Before Intervention Mean (SD)	After Intervention Mean (SD)	Before Intervention Mean (SD)	After Intervention Mean (SD)			
Cognitive flexibility	Response time (trails requiring no task switching), ms	1152.44 (181.10)	1002.51 (150.50)	1127.04 (200.76)	1140.37 (493.35)	0.168	0.558	0.102
	Response time (trials requiring task switching), ms	1429.03 (284.20)	1316.09 (247.07)	1483.10 (322.77)	1428.94 (317.61)	0.003 **	0.431	0.264
	Response time (trials requiring task switching—trails requiring no task switching), ms	276.60 (152.81)	313.58 (179.53)	356.05 (184.96)	288.57 (415.21)	0.759	0.734	0.298
	Accuracy (trails requiring no task switching), % correct	85.72 (11.76)	89.70 (8.31)	81.25 (13.78)	82.01 (16.72)	0.221	0.174	0.400
	Accuracy (trials requiring task switching), % correct	73.77 (19.52)	83.30 (11.97)	71.67 (17.19)	77.20 (18.02)	0.033 *	0.435	0.557

Values under time effect, group effect, and interaction have *p* values. * *p* < 0.05, ** *p* < 0.01.

## Data Availability

The data presented in this study are available on request the corresponding author.
